# The Effect of Synovial Fluid Enzymes on the Biodegradability of Collagen and Fibrin Clots

**DOI:** 10.3390/ma4081469

**Published:** 2011-08-22

**Authors:** Matthew Palmer, Elizabeth Stanford, Martha M. Murray

**Affiliations:** Department of Orthopaedic Surgery, Children’s Hospital of Boston, Harvard Medical School, 300 Longwood Ave, Boston, MA 02115, USA; E-Mails: matthew.palmer@gmail.com (M.P.); stanford.elizabeth@gmail.com (E.S.)

**Keywords:** collagen, fibrin, scaffold, fibroblast, enzyme

## Abstract

Recently there has been a great deal of interest in the use of biomaterials to stimulate wound healing. This is largely due to their ability to centralize high concentrations of compounds known to promote wound healing at a needed location. Joints present a unique challenge to using scaffolds because of the presence of enzymes in synovial fluid which are known to degrade materials that would be stable in other parts of the body. The hypothesis of this study was that atelocollagen scaffolds would have greater resistance to enzymatic degradation than scaffolds made of gelatin, fibrin and whole blood. To test this hypothesis, collagen and fibrin-based scaffolds were placed in matrix metallopeptidase-1 (MMP-1), elastase, and plasmin solutions at physiologic concentrations, and the degradation of each scaffold was measured at varying time points. The atelocollagen scaffolds had a significantly greater resistance to degradation by MMP-1, elastase and plasmin over the fibrin based scaffolds. The results suggest that atelocollagen-based scaffolds may provide some protection against premature degradation by synovial fluid enzymes over fibrin-based matrices.

## 1. Introduction

Recently there has been a great deal of interest surrounding the use of biomaterials as provisional scaffolds to stimulate the healing of tissues within joints. Targeted tissues include the anterior cruciate ligament (ACL) [1,2,3] and meniscus [4,5,6]. The biomaterials are designed to act as a substrate that allows cells to grow into, remodel, and eventually form new tissue [2,6]. Collagen scaffolds are reasonable biomaterials because of their ability to centralize high concentrations of compounds known to promote wound healing at a needed location [1,2]. Atelocollagen scaffolds are manufactured by using a pepsin solution to remove the antigenic ends of the collagen molecule, neutralizing the collagen, and lyophilizing to form a porous scaffold. Gelatin scaffolds are manufactured by lyophilizing a 1 to 2% weight by volume gelatin (hydrolysed collagen) suspension, also resulting in a porous scaffold. However, it is currently unknown how these different processing techniques will affect the function of the collagen scaffold as a biomaterial. Fibrin scaffolds and blood clot have also commonly been reported for use in enhancing ligament [7] and meniscal healing [6,8] with mixed reports of efficacy. Both collagen and pepsin can be cross-linked in order to increase their mechanical properties and control their degradation rate [9,10].

Joints present a unique challenge to using scaffolds because of the presence of enzymes in synovial fluid which are known to degrade materials that would be stable in other parts of the body [11,12,13,14]. Synovial fluid is composed of hyaluronan and D-N-acetylglucosamine, as well as many enzymes [15,16,17], among them matrix metallopeptidase 1 (MMP-1), elastase and plasmin, found in the synovial fluid at varying concentrations [18,19,20]. These three enzymes are hypothesized to affect the healing of intra-articular injuries [21,22,23,24,25,26,27,28]. MMP-1 is known to be involved with the breakdown of the extracellular matrix [21,22,23], as well as with tissue remodeling. It specifically degrades type I, II, and III collagen. Elastase is a peptidase that breaks down elastin and collagen fibers [19,24,25]. Plasmin is an enzyme that is present in synovial fluid in increased amounts after injury and degrades fibrin clots [26,27,28].

How these enzymes affect atelocollagen, gelatin, fibrin and blood-based scaffolds is critical to define if these materials are to be used in joint tissue engineering. The knee is one of many synovial joints found in the human body and it is vital to understand how a provisional scaffold will behave when placed in the synovial fluid [2]. By subjecting these materials to the concentration of enzymes found in synovial fluid (Table 1), this response can be studied. The structural integrity of the scaffolds is important when determining the ability of these scaffolds to withstand the joint environment long enough to participate in ACL repair or repair of other joint injuries. In addition, determining what effect, if any, the collagen structure will have on cellular proliferation within the scaffold could be useful for design of collagen-based scaffolds.

**Table 1:** Enzyme concentrations found in the synovial fluid of the knee and those used in this experiment.

Enzyme	Concentration in the knee	Experimental Concentration
MMP-1	177 to 279 µg/mL (Gysen, 1985) [18]	250 µg/mL
Elastase	0.041 to 101.8 µg/mL (Kleesiek, 1986) [19]	100 µg/mL
Plasmin	23 to 55 µg/mL (Kummer, 1992) [20]	40 µg/mL

In this study, we evaluated identically sized scaffolds of atelocollagen, gelatin, fibrin and blood clot biomaterials for their ability to withstand degradation by enzymes found in synovial fluid. We hypothesized that the collagen formulation—*i.e*., atelocollagen *vs.* gelatin—as well as the composition of the scaffold, would significantly affect the ability of the scaffold to facilitate cell proliferation and to resist degradation by enzymes found in the synovial fluid.

## 2. Results and Discussion

### 2.1. MMP-1 Degradation

**Figure 1:**
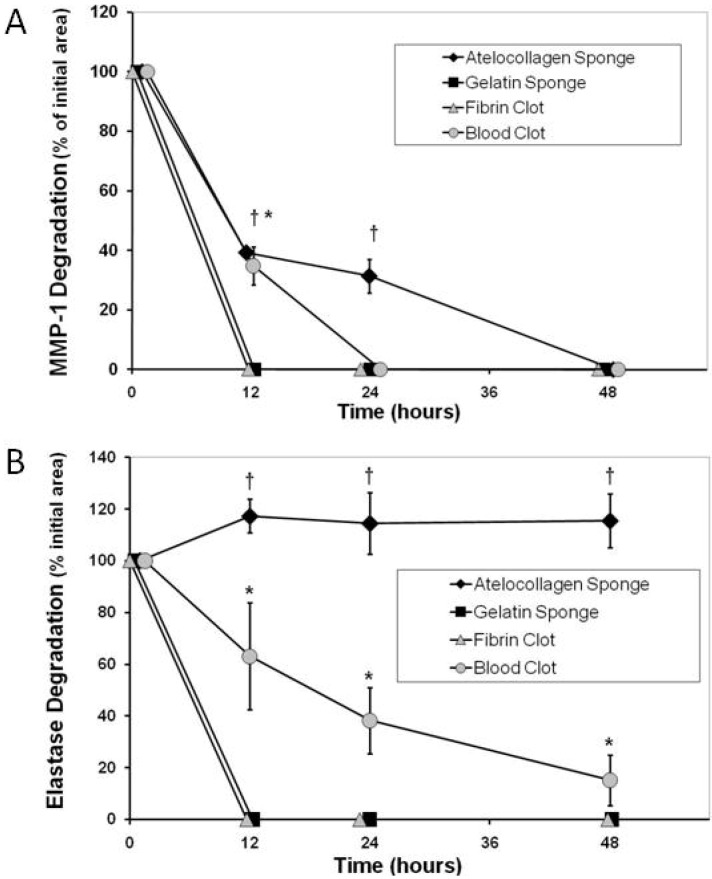

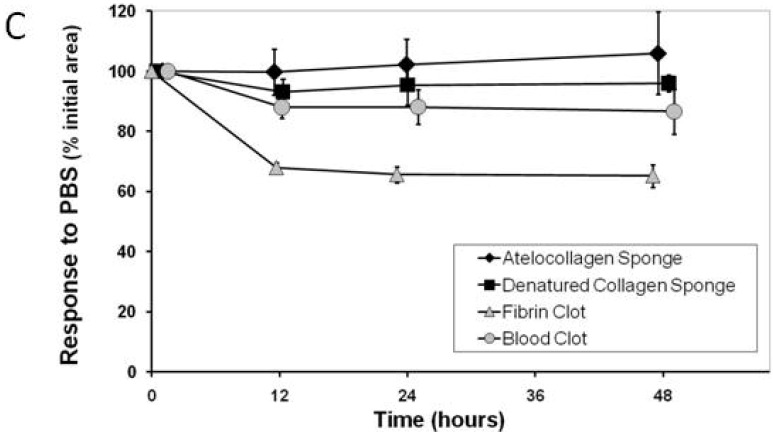
Enzymatic degradation of atelocollagen, gelatin, fibrin, and blood clot scaffolds over a 48 h period by (**A**) MMP-1; (**B**) Elastase and (**C**) Phosphate buffered saline (PBS) (control). By 24 h in the MMP-1 solution, the only remaining scaffolds with any integrity were the atelocollagen scaffolds and by 48 h, even the atelocollagen scaffolds had been degraded. † denotes significantly less degradation of the atelocollagen scaffolds at 12, and 24 h (p < 0.02). * denotes significantly less degradation of the blood clot scaffold than the fibrin or gelatin at the time points identified (p < 0.001 for all comparisons).

The atelocollagen scaffolds had a significantly higher resistance to degradation by MMP than all other groups at 12 and 24 h (Figure 1, p < 0.02 for all comparisons). While the blood clots still retained almost 40% of their area at 12 h, the gelatin and fibrin clot scaffolds were completely degraded after 12 h. After 24 h in the MMP-1 solution, the only remaining scaffolds with any integrity were the atelocollagen scaffolds, which had retained 39% of their area, and by 48 h, even the atelocollagen scaffolds had been degraded (Figure 1A).

### 2.2. Elastase Degradation

The atelocollagen scaffolds had significantly greater resistance to elastin than any of the other scaffolds (p < 0.001 for all comparisons, Figure 1B). Interestingly, the atelocollagen scaffolds exhibited complete resistance to degradation by the elastase solution throughout the experiment. In fact, in this group, there was an increase in scaffold area in the first 12 h (117 ± 7%) which remained at 48 h. The gelatin and fibrin clot scaffolds were completely degraded after 12 h (Figure 1B). The blood clot scaffolds were 63 ± 21%, 38 ± 13% and 15 ± 10% of their original sizes at 12, 24 and 48 h, respectively, having degraded significantly more than the atelocollagen scaffolds at all time points (p < 0.001).

### 2.3. PBS Degradation

Phosphate buffered saline (PBS) was the carrier used to deliver the MMP-1 enzyme and the elastase enzyme, and thus was also evaluated for its potential to influence scaffold degradation. The atelocollagen and gelatin scaffolds had minimal change in size in PBS. At 48 h, the atelocollagen scaffolds were 106 ± 14% and the gelatin scaffolds 96 ± 3% of their respective initial areas. The mean area of the blood clot and the fibrin clot scaffolds decreased, respectively, to 88 ± 4% and 68 ± 2% of their initial sizes at 12 h and remained stable throughout the remaining time of the experiment (Figure 1C).

### 2.4. Plasmin Degradation

**Figure 2:**
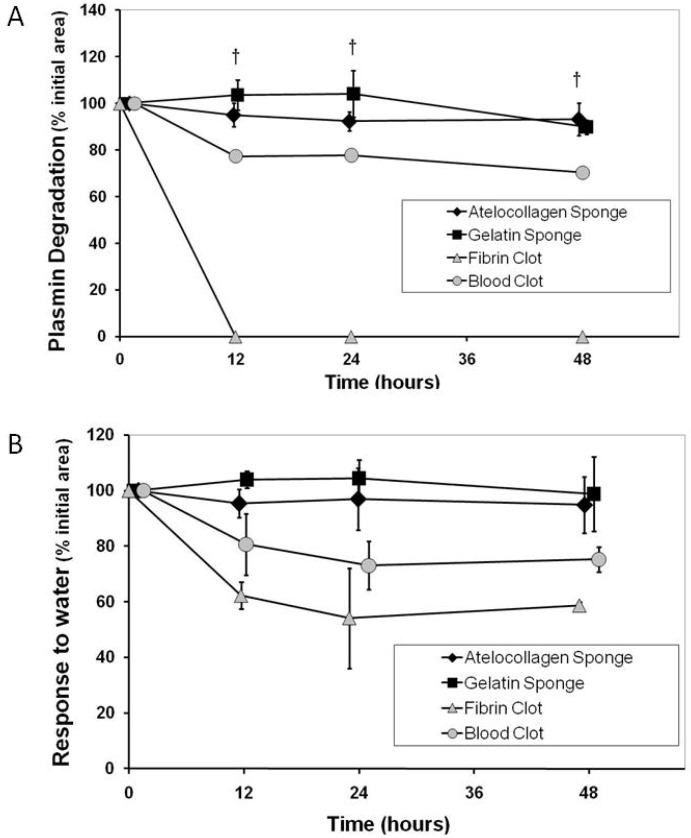
Enzymatic degradation of atelocollagen, gelatin, fibrin, and blood clot scaffolds over a 48 h period by (**A**) plasmin and (**B**) water (control). † denotes significantly less degradation by plasmin of atelocollagen and gelatin scaffolds than the blood clot or fibrin clot (p < 0.05 for all comparisons).

Both atelocollagen and gelatin scaffolds had improved resistance to plasmin degradation over the fibrin and blood clot scaffolds (Figure 2A, p < 0.02 for all comparisons). The two collagen scaffolds had similar plasmin resistance, with no significant difference in area between these groups at any time point (Figure 2A; p > 0.05). Blood clot scaffolds were significantly smaller than both the atelocollagen and gelatin scaffolds at all time points (p ≤ 0.02); whereas the fibrin clot scaffolds were completely degraded at 12 h.

### 2.5. Water Degradation

Water was the carrier used to deliver the plasmin enzyme, and thus was also evaluated for its potential to influence scaffold degradation. The atelocollagen and gelatin scaffolds exhibited slight decrease in size when subjected to water, with 95 ± 10% and 99 ± 14% of their respective initial areas at 48 h. The mean area of the blood clot and the fibrin clot scaffolds decreased, respectively, to 88 ± 11% and 62 ± 5% of their initial sizes and remained stable throughout the remaining time of the experiment (Figure 2B).

### 2.6. Cell Proliferation

**Figure 3:**
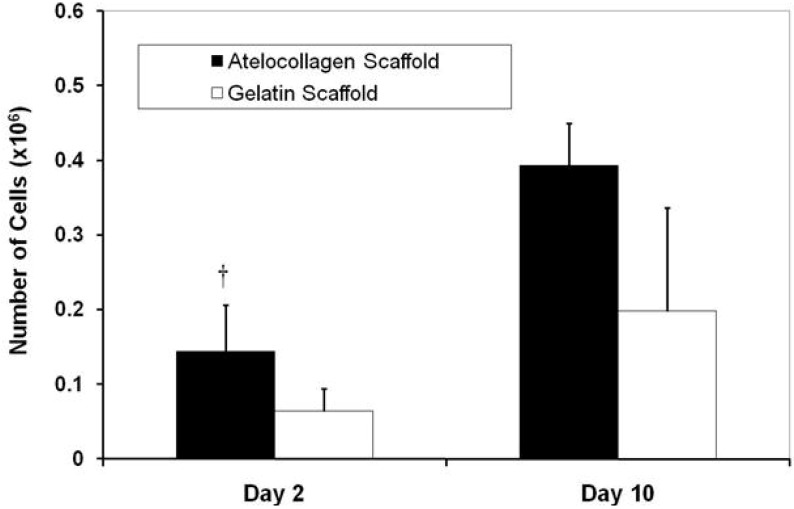
Cell proliferation within atelocollagen and gelatin scaffolds at 2 and 10 days after seeding as measured by the MTT assay. Cell numbers in both types of scaffolds increased between 2 and 10 days. Atelocollagen scaffolds had a higher cell concentration at 2 days. † denotes significantly higher cell number in the atelocollagen scaffolds at 2 days (p < 0.05).

The atelocollagen scaffolds had more cells present than the gelatin scaffolds at 2 days (0.14 × 10^6^ ± 0.03 × 10^6^ *vs.* 0.06 × 10^6^ ± 0.06 × 10^6^ cells) and 10 days (0.39 × 10^6^ ± 0.14 × 10^6^ *vs.* 0.20 × 10^6^ ± 0.06 × 10^6^ cells), p < 0.05 for both days. The atelocollagen and gelatin scaffolds experienced 2.7 ± 1.1 and 3.1 ± 3.2 times increase in cell number, respectively, from day 2 to day 10 (p = 0.40; Figure 3).

### 2.7. Scaffold Contraction

After 9 days in culture, the cell-seeded atelocollagen scaffolds had contracted more than the cell-seeded gelatin scaffolds (91.7 ± 5.2% of day 1 surface area *vs.* 101.1 ± 8.5% of day 1 surface area, p < 0.05). Control scaffolds, which had been cultured without cells, showed similar contraction rates to those seen in the cell-seeded scaffolds (88.3 ± 7.0% of day 1 surface area *vs.* 101.1 ± 8.5% of day 1 surface area, p = 0.06).

**Figure 4:**
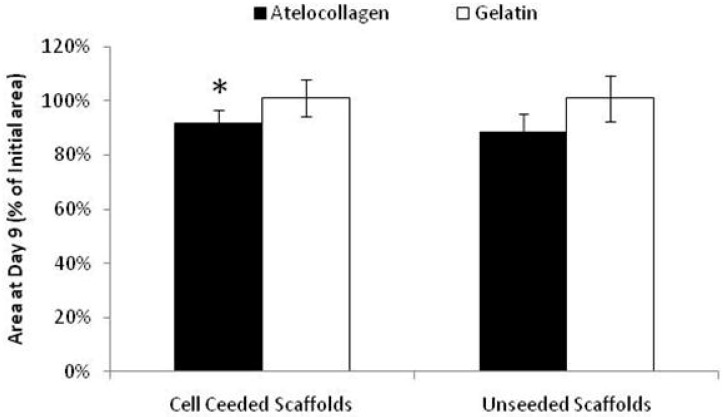
Contraction of scaffolds cultured for 9 days measured as a percentage of initial area. The atelocollagen scaffolds had a greater reduction in surface area than the gelatin scaffolds. A similar trend was seen for cell seeded scaffolds, and non-cell seeded scaffolds. * denotes significantly greater contraction of the cell seeded atelocollagen scaffold compared to the cell seeded gelatin scaffold (p < 0.05).

## 3. Experimental Section

### 3.1. Manufacturing of Atelocollagen Scaffolds

The collagen used in this study was derived from bovine knees (Research 87, Boylston, MA, USA). The tissue was sterilely harvested, minced, and solubilized in an acidic pepsin solution. The resulting slurry was frozen overnight at −80 °C and lyophilized under full vacuum at −35 °C for three days and re-hydrated with sterile water to a collagen concentration >10 mg/mL. The same reconstituted atelocollagen slurry was used in all experiments.

The atelocollagen scaffolds were then made by mixing the atelocollagen slurry with HEPES Buffer (Cellgro, Mediatech, Inc, Herndon, VA, USA), and neutralizing to a pH of 7.4 using 7.5% sodium bicarbonate (Cambrex BioScience Walkersville, Inc., Walkersville, MD, USA). The solution was transferred to a sterile 100 mm petri dish, and incubated for 20 min at 37 °C and 5% CO_2_ to allow for gelation. The resulting gel was frozen at −80 °C overnight and lyophilized.

### 3.2. Gelatin Scaffolds

Commercially available gelatin scaffolds (Surgifoam® sheets, Johnson and Johnson, Binghampton, NY, USA) of the same thickness of the atelocollagen sheet were obtained, and a sterile one centimeter diameter circular punch was used to cut out individual scaffolds from both materials. Unlike the atelocollagen scaffolds which were not cross-linked, the gelatin scaffolds were chemically cross-linked with an aldehyde during manufacturing.

### 3.3. Preparation of Fibrin Clot Scaffolds

Fibrin clots were prepared by adding a 500 units/mL thrombin NaCl solution (King Pharmaceuticals, Bristol, TN) to complete medium (Dulbecco’s Minimum Essential Medium (DMEM) (Cellgro, Mediatech Inc., Herndon, VA, USA) supplemented with 10% defined fetal bovine serum (FBS) (Hyclone, Logan, UT) and 1% antibiotic-antimycotic solution (Cellgro, Mediatech Inc., Herndon, VA, USA), to create a 10 units/mL solution. 715 μL of the thrombin solution was transferred to each well of a 24-well plate. 285 μL of a 20 mg/mL fibrinogen solution (Sigma-Aldrich, Saint Louis, Missouri, Product#: F4753) was added to each thrombin aliquot. The plate was mixed on a shaker table at 60 oscillations/min for 30 seconds. The plate was put into an incubator (37 °C, 5% CO_2_) for 30 min to allow fibrin clot formation. A sterile one centimeter circular punch was then used to cut individual scaffolds.

### 3.4. Preparation of Blood Clot Scaffolds

A total of 300 mL of whole blood was drawn from two hematologically normal volunteers meeting all criteria of the American Association of Blood Banks (Food and Drug Administration, Center for Biologics Evaluation and Research). Blood was collected in 60 cc syringes with 10% acid-citrate dextrose as an anticoagulant at the Immune Disease Institute (Boston, MA, USA). 120 mL of blood was used for making blood clots while 180 mL was used for making platelet-rich plasma as described in the next section. A solution of 1000 Units thrombin/mL CaCl_2_ was used to clot the whole blood. 100 μL aliquots of the thrombin solution were placed into wells of a 24-well plate. 900 μL of whole blood was added, and the plate was placed on a shaker table at 60 oscillations/minute for 30 seconds, and then incubated (37 °C, 5% CO_2_) for 20 min to complete blood clot scaffold formation. A sterile one centimeter circular punch was then used to create uniform individual scaffolds.

### 3.5. Preparation of Enzyme Stock Solutions

The MMP-1 stock solution was made by combining 50 mg of MMP-1 (Worthington Biochemical Corporation, Lakewood, NJ, product number LS004214) with 200 mL of PBS, yielding a 250 µg/mL (72.2 units/mL) MMP-1 solution. Aliquots of the solution were then frozen at −80 °C in 50 cc tubes.

The elastase stock solution was made by combining 5 mg of elastase (Worthington Biochemical Corporation, Lakewood, NJ, product number LS006363) with 50 mL of PBS, yielding a 100 µg/mL (0.86 units/mL) solution. Aliquots of the solution were then frozen at −80 °C in 50 cc tubes.

The plasmin stock solution was made by combining 1.5 mg of plasmin (Sigma Aldrich Co., St. Louis, MO, USA, product number P1867) with 37.5 mL of ultrapure water to yield a 40 µg/mL plasmin (0.416 units/mL) solution. Aliquots of the solution were then frozen at −80 °C in 50 cc tubes.

### 3.6. Enzyme Degradation Experiment

Enzymatic degradation rate was determined by measuring the change in surface area of the scaffolds over time. Atelocollagen, gelatin, fibrin clot, and blood clot scaffolds were subjected to each of the enzyme stock solutions. Experiments were run in triplicate. We also observed the response of the scaffolds when exposed to the solvents used to make the enzyme stock solutions (PBS for MMP-1 and elastase; water for plasmin). The scaffolds were placed into wells of a 12-well plate and 1.0 mL of solution was added to each well. The solutions were changed after 12, 24, and 36 h. Digital images were taken with a Canon Rebel XT camera at 12 h, 24 h, and 48 h. Surface area measurements of the scaffolds were determined using the NIH *Image-J* software (v. 1.38×).

### 3.7. Cell Source

Human ACL explants were obtained from the knee using sterile technique during ACL reconstruction. After ligament harvest, explants were cultured in completed medium (Dulbecco’s modified Eagle medium or DMEM) containing 4.5 g/L glucose, 10% fetal bovine serum, and 1% AB/AM) which was changed two times per week. When primary outgrowth cells were 80% confluent, they were trypsinized, counted, and suspended in complete medium.

### 3.8. Measurement of Cell Proliferation in Scaffolds

Cell proliferation was determined by indirectly measuring the change in cell number in the scaffolds between two and ten days, using the MTT (3-(4,5-dimethylthiazol-2-yl)-2,5-diphenyltetrazolium bromide) assay. Atelocollagen and gelatin scaffolds (n = 6, per experimental group and time point) were placed into the wells of 12-well plates. The scaffolds were seeded with 2.5 × 10^5^ cells by placing 250 μL of the cell solution on top of each scaffold and incubating (37 °C, 5% CO_2_) for one hour to allow for absorption. After incubation, 0.75 mL of complete medium was added to each well and the plates were placed back into the incubator. Media was changed every other day.

At 2 and 10 days, the MTT working solution was prepared at a concentration of 1 mg/mL in complete medium from a sterile stock MTT solution (Sigma-Aldrich, St. Louis, MO, USA, Cat. #: M5655-500MG). The media was aspirated from each well and 1 mL of the working MTT solution was added to each well. The plates were incubated for 3 h (37 °C, 5% CO_2_). Subsequently, the excess MTT solution was removed and 1 mL of sterile 1X Phosphate Buffer Saline (EMD Chemicals, Gibbstown, NJ, USA, Cat. #: B10241-34) was added to each well, placed on a agitator table (Fisher Scientific Clinical Rotator, 100 rpm) and left to rinse at room temperature for 30 min. Rinses were repeated until the absorbance readings of the wash were less than 0.100. All PBS was then removed and each scaffold transferred into 3 ml centrifuge tubes. 1mL of a detergent containing 20% aqueous SDS/formamide (1:1 volume ratio) was added to each tube and incubated overnight in a 37 °C water bath. The tubes were vortexed for 5 seconds and then centrifuged for 5 min at 250 × g. Aliquots of the supernatant from each tube (200 µL) were then transferred into a 96-wellplate, and the absorbencies were measured at 562 nm. The cell proliferation assay was carried out after 2 and 10 days of culture, and cell proliferation was determined by calculating the change in absorbance from day 2 to day 10.

Measurement of the contraction of the atelocollagen and gelatin cell-seeded scaffolds was also performed using surface area measurements of the scaffolds at days 1, 2, 5, 6, 7 and 9. Photos of the scaffolds were taken and the area of each scaffold calculated using *Image J*. Contraction was recorded as the percent change in scaffold area compared to day 1.

### 3.9. Statistical Analysis

All statistical analysis was performed using SPSS version 16.0. For the enzyme degradation experiments, a mixed model repeated measures ANOVA was utilized. For the cellular proliferation experiments, a two way ANOVA was used with Fisher’s LSD *post hoc* test to detect significant differences between groups. Results were given as mean ± SD. For all experiments, a p-value < 0.05 was deemed statistically significant.

## 4. Discussion

It has been found that after trauma to a joint occurs, the concentration of certain enzymes in synovial fluid increases. This release of enzymes can lead to more rapid degradation of connective tissue, as well as degradation of scaffolds used for tissue repair in the joint environment. When using a scaffold for tissue repair, it is important that the scaffold not dissolve before the surrounding cells can invade and stabilize it [29,30,31]. The results here suggest that scaffolds made of atelocollagen may be better able to resist degradation when compared to gelatin scaffolds or fibrin clots.

This result may help to reconcile several seemingly contradictory reports on the use of biologically based scaffolds for joint repair. The use of platelet-rich plasma products, which are fibrin-based, has not demonstrated positive results when used in the synovial environment for ACL repair [32] or rotator cuff healing [33]. In contrast, when the fibrin-based materials are used outside of the joint environment, they appear to have greater success [7]. For example, use of PRFM on the synovial side of a rotator cuff tendon tear (which is exposed to synovial fluid) has been found to be ineffective [33], whereas the use of a different fibrin-based product on the bursal side of the tendon (outside of the joint) has shown early efficacy [34]. In addition, animal studies have demonstrated that while the use of fibrin based scaffolds alone is ineffective in ACL repair [32] and intra-articular rotator cuff repair [33], the use of collagen-based scaffolds is effective in stimulating ACL repair [35,36,37] and rotator cuff repair [38,39].

The fibrin and blood clot scaffolds decreased in size during the first 12 h of culture in PBS and water, and then had no significant further change. Due to the lack of further change on exposure to these solutions, it is unlikely these initial decreases in size were due to degradation of the scaffolds; rather it is more likely these changes were due to an initial contraction of the scaffolds. In the case of the blood clot scaffolds, this contraction may have been due to the presence of platelets in the scaffold as platelets are known to have a contractile function [40,41]. For the fibrin scaffolds, the reason for the initial contraction is less clear. Improved organization of the fibrin fibrils is one possibility [42]. Taking into account this initial contraction, the difference between the change in size in the carrier and that in the enzyme solutions is likely due to degradation.

One of the major limitations with all of the scaffolds tested here was their degradation in the MMP-1 solution by 48 h. The length of time required for a scaffold to be present in a wound site before it is stabilized sufficiently by invasion of surrounding cells is as yet unknown. Previous studies have demonstrated that immature animals have a more rapid cellular invasion of an ACL wound site than adult animals [43], likely due to the observed increased cellular proliferation and migration speed in these animals when compared with adults [44,45]. Thus, one possible reason for the improved functional ACL healing noted in immature animals [35] may be that the immature cells are able to stabilize the atelocollagen implants before the synovial enzymes degrade it, while the slower adult cells are not able to populate the scaffold before it degrades. Use of a scaffold material with greater resistance to synovial degradation may be necessary to obtain functional healing in the adult knee. Another strategy would be to remove or neutralize the enzymes in the adult animals. Arthroscopy results in washing out of the enzymes and replacement with saline, and this act alone may help tip the balance in favor of scaffold preservation. What effect an additional synovectomy may have on the re-accumulation of the enzymes is also as yet unknown, but may be a key factor in the success of biologic scaffolds.

The atelocollagen and gelatin had different manufacturing protocols which may have affected results. The gelatin is cross-linked during its production, while the atelocollagen is not. However, the addition of cross-linking should increase the resistance of the atelocollagen to enzymatic degradation [9,10], so if the atelocollagen were manufactured the same way as the gelatin, one might expect even greater resistance to degradation, not less.

Tissue engineering within the synovial joint is a challenging task. In this work, we define some of the basic material responses to elements that are intrinsic to this unique environment; namely, the presence of enzymatic agents that are not found in significant quantities in other tissues. The results suggest that atelocollagen-based scaffolds may provide some protection against premature degradation by synovial fluid enzymes over blood or fibrin clots, but even they may be degraded within 48 h by MMP-1 at high enough levels in the traumatized knee. Additional in vivo evaluations of these materials is likely to provide further insight into the use of these biologic materials in tissue engineering within the joint.

## 5. Conclusions

The atelocollagen scaffolds had a significantly greater resistance to degradation by MMP-1, elastase and plasmin over the fibrin based scaffolds; while the cellular proliferation was similar in both types of collagen scaffolds. The atelocollagen scaffolds contracted more than the gelatin scaffolds during 9 days in culture, though this does not appear to be cell mediated, as similar results were seen for the non-cell seeded scaffolds. The results suggest that atelocollagen-based scaffolds may provide some protection against premature degradation by synovial fluid enzymes over fibrin-based matrices.
